# The complement system, neuronal injury, and cognitive function in horizontally-acquired HIV-infected youth

**DOI:** 10.1007/s13365-016-0460-5

**Published:** 2016-06-06

**Authors:** Jennifer L. McGuire, Alexander J. Gill, Steven D. Douglas, Dennis L. Kolson, CNS HIV Antiretroviral Therapy Effects Research (CHARTER) Group

**Affiliations:** 1 Division of Neurology, The Children’s Hospital of Philadelphia, 34th St and Civic Center Blvd, Philadelphia, PA 19104 USA; 2 Department of Neurology, Perelman School of Medicine, University of Pennsylvania, Philadelphia, PA USA; 3 Department of Pediatrics, Perelman School of Medicine, University of Pennsylvania, Philadelphia, PA USA; 4 Division of Allergy and Immunology, The Children’s Hospital of Philadelphia, Philadelphia, PA USA; 5 The Children’s Hospital of Philadelphia Research Institute, Philadelphia, PA USA

**Keywords:** HIV, HAND, Neurofilament, Complement, Neuroinflammation, Cognition, Youth

## Abstract

The complement system (C1q/C3) is a key mediator of synaptic pruning during normal development. HIV inappropriately induces C1q and C3 production in the brain, and reduces neuronal complement inhibition. HIV may thus alter neural connectivity in the developing brain by excessively targeting synapses for elimination. The resultant pattern of neuronal injury may fundamentally alter neurodevelopmental and cognitive processes differentially across ages. This study aimed to (1) measure the association between the cerebrospinal fluid (CSF) complement factors (C1q/C3) and a marker of neuronal injury (NFL) in HIV+ subjects; (2) quantify the differences in CSF C1q/C3 between HIV+ youth and older adults; and (3) define the relationship between CSF C1q/C3 and cognitive impairment in each age group. We performed a retrospective cross-sectional study of 20 HIV+ 18–24-year-old youth and 20 HIV+ 40–46-year-old adults with varying levels of cognitive impairment enrolled in the CNS Antiretroviral Therapy Effects Research study. We quantified C3, C1q, and NFL by ELISA in paired CSF/plasma specimens. We found that CSF C1q correlates with NFL in all subjects not receiving antiretroviral therapy (*n* = 16, rho = 0.53, *p* = 0.035) when extreme NFL outliers were eliminated (*n* = 1). There was no difference in plasma/CSF C1q or C3 between older adults and youth. In 18–24-year-old youth, a nearly significant (*p* = 0.052) elevation of CSF C1q expression was observed in cognitively impaired subjects compared to cognitively normal subjects. Further investigation into the role of the CNS complement system in the neuropathogenesis of HIV is warranted and should be considered in a developmentally specific context.

## Introduction

The complement system is a key mediator of synaptic pruning during normal development (Stevens et al. 2007; Stephan et al. 2012), and when inappropriately activated later in life, has been implicated as one mechanism of synapse elimination in a variety of different neurodegenerative diseases (Alexander et al. 2008; Stephan et al. 2012). Complement factors are locally synthesized in the brain and are developmentally regulated in localized patterns. C1q and C3 appear to be particularly important in tagging weak or unnecessary synapses for removal via microglia complement receptor-mediated phagocytosis. Consistent with this function, C1q- and C3-deficient mouse models have large and persisting defects in central nervous system (CNS) synaptic connectivity (Stevens et al. 2007). Furthermore, C3 and CR3 knockout mice have more structural synapses in the visual system than wild-type counterparts, suggesting that altered complement signaling early in development results in sustained defects in synaptic connectivity (Schafer et al. 2012).

The role of the complement system in HIV has been extensively studied in the periphery. Complement is activated early in HIV infection via a variety of mechanisms, but it fails to eradicate HIV from infected cells and from the circulation due to the presence of complement regulators within the HIV-1 envelope and on infected cells (Liu et al. 2014). Instead, HIV uses complement activation to spread and enhance systemic infection (Liu et al. 2014). The role of complement in the neuropathogenesis of HIV is less well understood. Local HIV infection induces C3 production in neuronal and glial cell lines incubated with HIV and in parallel downregulates normal complement inhibitor proteins (CD59) (Stephan et al. 2012; Liu et al. 2014). In a simian immunodeficiency virus (SIV) model, cerebral synthesis of C1q and C3 was upregulated compared to controls and deposited on neuronal membranes (Speth et al. 2004). In human cerebrospinal fluid (CSF), C3 levels are elevated in HIV+ subjects with signs of CNS dysfunction compared to uninfected controls, as are C3 CSF:serum ratios in HIV+ subjects compared to uninfected controls (Jongen et al. 2000). In human neuropathologic studies, complement component upregulation was demonstrated in the neostriatum of individuals with HIV-encephalitis (Gelman et al. 2012), the traditional pathological correlate of HIV-associated dementia (HAD).

These HIV-specific observations of the complement system in the context of our emerging understanding of its role in normal developmental synaptic pruning raise several pertinent questions in the study of neuroHIV across the age span. First, does complement alter neural connectivity in HIV by excessively targeting synapses for elimination? And second, does any resultant pattern of neuronal injury fundamentally alter neurodevelopmental and cognitive processes differentially across ages depending on normal pre-existing complement developmental regulation? Approximately one quarter of the 50,000 new HIV infections in the USA per year are among 13–24-year-old youth (Centers for Disease Control and Prevention 2015). Normal neural circuit maturation continues from infancy into young adulthood (up to 30 years of age), requiring activity-dependent pruning of inappropriate synaptic connections in order to reinforce and strengthen appropriate connections (Giedd et al. 1999). It is therefore important to study and understand the age-specific effects of acquired HIV on the actively developing brain in order to tailor future ontogenetic-appropriate interventions and prevention strategies in neuroAIDS.

No published studies to date have examined the relationships between the CNS complement system and markers of neuronal injury in HIV infection. Neurofilament light chain (NFL) is a structural protein specific to neurons that is released into the CSF and blood following axonal disruption (Julien 1999; Pasol et al. 2010; Gresle et al. 2011). In HIV infection, CSF NFL levels appear to be an appropriate surrogate marker for neuronal damage. CSF NFL is elevated in early and later infection in subjects with and without cognitive impairment (Peluso et al. 2013; Jessen Krut et al. 2014), although levels are highest in HIV-associated dementia (HAD) (Gisslen et al. 2007; Abdulle et al. 2007; Mellgren et al. 2007) and fluctuate in response to combined antiretroviral therapy (increase with interruption and decrease with initiation) (Abdulle et al. 2007).

In order to begin to examine the role of the CNS complement system as one mechanistic player in HIV neuropathogenesis, we sought to elucidate the relationship between complement factors and neuronal damage in HIV infection across the developmental age span. Specifically, we measured the correlations among CSF and plasma complement factors (C1q and C3) and NFL as a marker of neuronal injury. We then examined the differences in complement protein concentrations in youth and older adults, and finally explored the relationship between these markers and cognitive function in each age group.

## Methods

### Study design and setting

We performed a retrospective cross-sectional study using biological samples (plasma, CSF) and data from 40 HIV+ subjects enrolled in the CNS HIV Antiretroviral Therapy Effects Research (CHARTER) cohort of the NIMH/NINDS/NIH. Characteristics of the CHARTER cohort are described elsewhere (Heaton et al. 2010). Briefly, CHARTER is an ongoing, observational cohort study that enrolled 1561 HIV-infected persons between 2003–2007 from six US university-affiliated HIV treatment centers. Inclusion criteria for the CHARTER study were broad, but individuals with severe comorbid psychiatric, medical, or neurological disorders deemed likely to adversely affect cognitive functioning were excluded. HIV-negative subjects were not included in CHARTER. Our study used data and samples from 40 CHARTER subjects (20 each 18–24 years old and 40–46 years old). The cohort included men and women of any race/ethnicity with a spectrum of cognitive dysfunction, on and off antiretroviral therapy (ART), and who underwent successful lumbar puncture, venipuncture, and neuropsychological testing. Subjects with known hepatitis C infection, a concurrent diagnosis of syphilis, active substance abuse, a history of CNS opportunistic infection, head trauma, epilepsy, multiple sclerosis, other known causes of autoimmune dysfunction, mental retardation or dementia, psychotic disorder, or other chronic illness were excluded.

### Data collection

Original data collection for the CHARTER cohort was approved by the Human Subjects Protection Committees of each participating institution. All subjects provided written consent to participate in the CHARTER study. Data were originally obtained through comprehensive neuromedical, neurocognitive, psychiatric, and functional evaluations, and collection of blood, urine, and CSF samples (Heaton et al. 2010). The de-identified data and biological samples for the present substudy were obtained with permission of the CHARTER steering committee. Because the dataset and samples were de-identified and because our substudy did not involve patient contact, The Children’s Hospital of Philadelphia institutional review board determined (June 22, 2015) that this study did not qualify as human subjects research.

### Laboratory assessments

HIV infection was diagnosed by ELISA with Western blot confirmation. Clinical laboratory assessments, including complete blood counts, chemistry panels, and flow cytometry for CD4+ T lymphocyte count were performed at each CHARTER site’s Clinical Laboratory Improvement Amendments (CLIA) certified, or CLIA equivalent, medical center laboratory. Plasma HIV RNA measurements (viral loads) were quantified by a RT-PCR ultrasensitive assay (nominal lower quantitation limit 50 copies per mL; Amplicor®, Roche Diagnostic Systems, Indianapolis, IN) in a central lab (Heaton et al. 2010).

Biomarkers for the present substudy were measured in triplicate by validated, commercially available 96-well plate ELISAs on stored, frozen samples (−80C). Paired CSF/plasma samples were assayed for C3 (Abcam, limit of detection 0.2 μg/mL [plasma, catalog number ab108822], 0.5 ng/mL [CSF, catalog number ab108823]) and C1q (Abcam catalog number ab170246, limit of detection 0.03 ng/mL). CSF samples were assayed for NFL (Uman Diagnostics AB, limit of detection 31 ng/mL). Previous work by our group did not demonstrate measurable quantities of NFL in plasma (McGuire et al. 2015) so this was not assayed in the present study.

### Neurocognitive assessments

All CHARTER study subjects completed a comprehensive neuropsychological test battery assessing verbal fluency, executive functioning, speed of information processing, learning, recall, working memory, and motor skills. Raw test scores were converted to demographically adjusted *T* scores using the best available normative data accounting for age, sex, ethnicity, and education. Functional impairment was assessed using the Patient’s Assessment of Own Functioning Inventory (PAOFI) and an instrumental activities of daily living (IADL) questionnaire (Heaton et al. 2010). A global performance score was determined as previously described (Carey et al. 2004; Woods et al. 2004). HIV-associated neurocognitive disorder (HAND) status was classified according to Frascati criteria (Antinori et al. 2007).

### Data analysis and statistical methods

Data were analyzed using Stata version 14.0 (StataCorp, College Station, Texas, 2015). Non-parametric analysis methods were used given small sample sizes to improve accuracy of statistical estimates and reduce sensitivity of analyses to any statistical outliers. Continuous variables were described using median and intraquartile range (IQR), and intergroup differences were evaluated using the Wilcoxan rank-sum tests and Kruskal-Wallis tests. Categorical variables were described using counts and percents, and intergroup differences were compared using the Chi-square test. Spearman’s correlation coefficients were used for correlations between biomarkers. Statistical significance was determined a priori as a two-tailed *p* value <0.05.

## Results

Cohort demographics, clinical, and laboratory characteristics of everyone and of each specific age group are summarized in Table 1. Among the 40 total subjects examined, 88 % were male and 33 % were African American. As expected, older adults had a longer duration of infection (median 8.3 years versus 1.2 years, *p* = 0.005), and a subsequent lower nadir CD4+ T lymphocyte count (median 109 versus 298 cells/mm^3^, *p* = 0.020). Other markers of systemic infection (HIV-1 RNA viral load, current CD4+ T lymphocyte count) were similar between the different age groups. Older adults trended towards more frequent current ART use (70 % versus 45 %, *p* = 0.053), likely related to duration of time linked into appropriate care and counseling. Younger adults had higher Wide Range Achievement Test (WRAT) scores (102 versus 90, *p* = 0.025), suggesting a higher level of premorbid cognitive functioning despite comparable total years of education. It is unclear if this difference is simply reflective of the population currently being infected with HIV, or if it is age-related. However, all neurocognitive data are adjusted for age and demographic factors, making the latter possibility less likely. HAND diagnoses were comparable between the two age groups. Finally, no subjects were using illicit drugs at the time of this study.

**Table 1:** Demographic, HIV, and cognitive characteristics of the study population

Characteristics^a^	All *n* = 40	Youth (18–24 yearsold) *n* = 20	Older adults (40–46 years old) *n* = 20	*p* value^b^
Demographic variables				
Male sex	35 (88)	18 (90)	17 (85)	0.633
Black race	13 (33)	6 (30)	7 (35)	0.986
Age (years)	32 (22–42)	22 (20.5–23.5)	42 (40–43.5)	<0.001
HIV variables				
Duration of HIV (years)	3.1 (0.5–11.2)	1.2 (0.3–3.1)	8.3 (3.3–14.1)	0.005
Currently using ART	23 (58)	9 (45)	14 (70)	0.053
CD4 nadir	233 (59–410)	298 (199–521)	109 (35–356)	0.020
Plasma CD4+ count: 50–199	6 (15)	2 (10)	4 (20)	0.587
200–349	10 (25)	6 (30)	4 (20)	–
≥ 350	24 (60)	12 (60)	12 (60)	–
Plasma HIV-1 RNA (log)	2.96 (1.70–3.90)	2.96 (1.60–4.23)	2.96 (1.70–3.55)	0.815
CSF HIV-1 RNA (log)	1.70 (1.70–2.36)	1.70 (1.60–2.71)	1.70 (1.70–2.24)	0.750
Cognitive variables				
Education (years)	12 (11–13)	12 (11.5–13)	13 (10.5–14.5)	0.335
WRAT score	93.5 (83–108)	102 (90–112)	90 (76–100)	0.025
HAND normal	17 (43)	7 (35)	10 (50)	0.548
ANI, *n*(%)	17 (43)	10 (50)	7 (35)	–
MND, *n*(%)	1 (3)	0 (0)	1 (5)	–
HAD, *n*(%)	1 (3)	1 (5)	0	–
Not classified	4 (10)	2 (10)	2 (10)	–

^a^Categorical variables are described using *n*(%). Continuous variables are described using median (IQR)

^b^ *p* values to compare characteristics between age groups were calculated using chi-square tests for categorical variables and the Wilcoxan rank-sum (Mann-Whitney *U*) tests for continuous variables

**Fig. 1:**
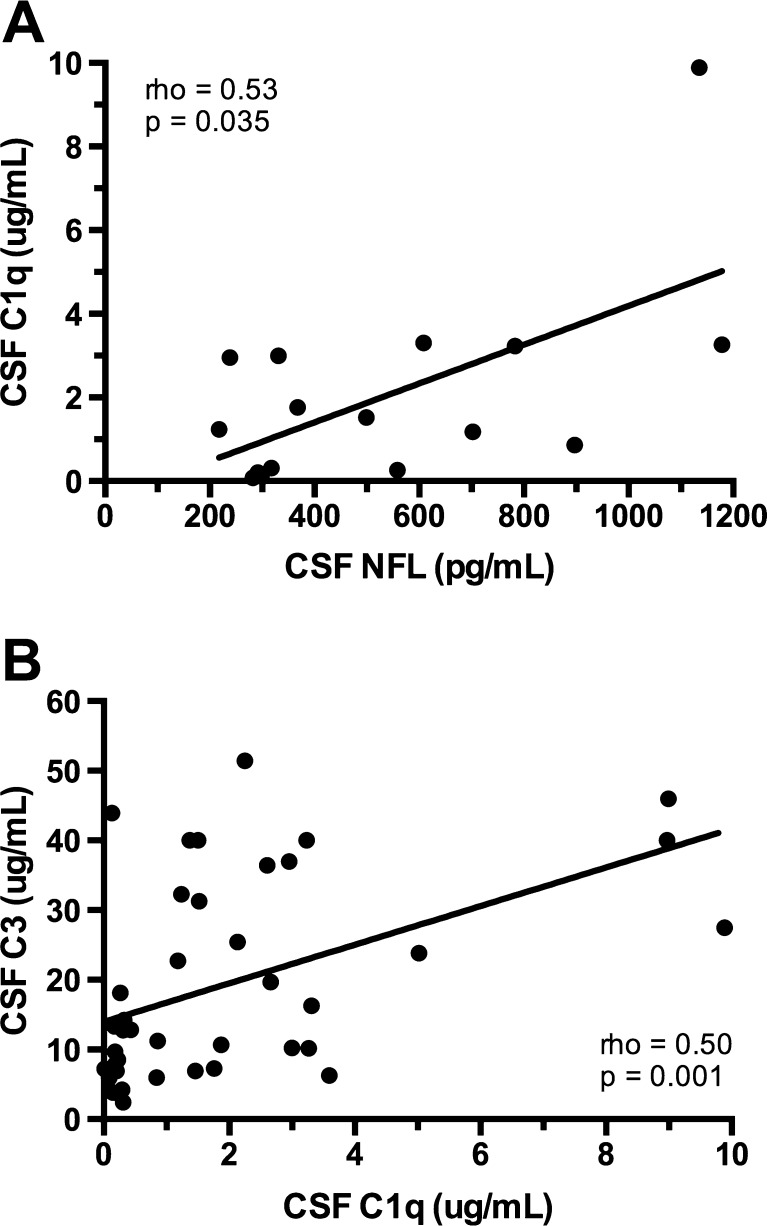
**a** There is a positive correlation between CSF C1q and NFL in subjects of all ages not currently receiving antiretroviral therapy (one outlier >3 SD removed). **b** There is a positive correlation between C1q and C3 in CSF. Correlations were analyzed using Spearman’s correlation coefficients

To examine the association between CSF complement proteins and neuronal injury in HIV, we first measured CSF expression of C1q/C3 and NFL. Analyses were conducted 1) with the entire cohort, and 2) restricted to subjects not receiving ART in order to avoid any potential confounding effects of ART on expression of biomarkers of neuroimmune dysregulation or neuronal injury. There were no significant correlations between NFL and either CSF complement marker in the overall cohort. However, the correlation between CSF C1q and NFL in subjects off ART (*n* = 17) did approach statistical significance (rho = 0.338, *p* = 0.184). We subsequently performed an exploratory analysis eliminating outlier NFL values (defined by value ≥3SD above median) that demonstrated a significant relationship between CSF C1q and NFL (*n* = 16, rho = 0.53, *p* = 0.035) (Fig. 1a). These findings suggest that complement may be linked to neuronal injury. Whether this is a parallel finding in the neuropathogenesis of HIV or causal is unclear in this cross-sectional analysis. Furthermore, both CSF C1q and C3 correlate closely with each other (rho = 0.5, *p* = 0.001) (Fig. 1b), suggesting these data are likely demonstrating true differences in activation of the complement system.

**Fig. 2:**
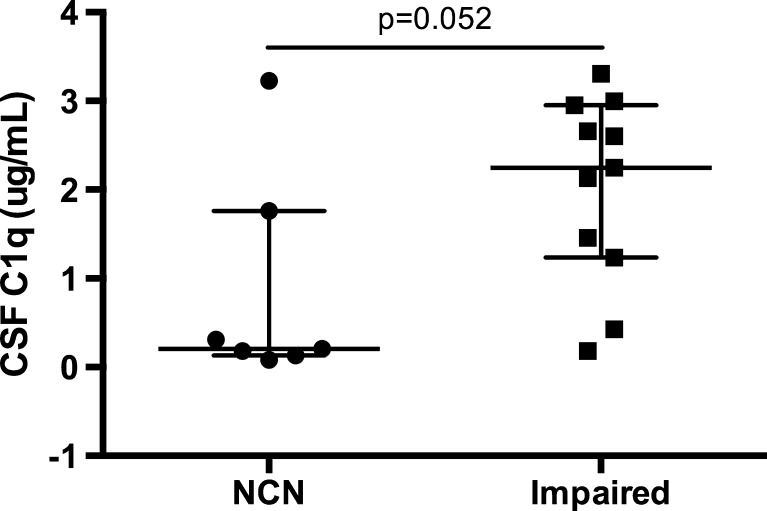
In 18–24 year-old youth, C1q was higher in cognitively impaired subjects compared to neurocognitively normal (NCN) subjects (median 2.13 versus 0.86ug/mL, *p* = 0.052)

To determine whether the proposed relationship between CSF C1q and NFL may be clinically significant, we next compared expression of CSF complement factors with the presence or absence of cognitive impairment (defined as meeting Frascati criteria for asymptomatic neurocognitive impairment [ANI], mild neurocognitive disorder [MND], or HIV-associated dementia [HAD]). Of note, four of the 40 total subjects were not classified for HAND so were therefore excluded from this analysis. In 18–24 year-old youth, a nearly significant (*p* = 0.052) elevation of CSF C1q expression was observed in cognitively impaired subjects compared with cognitively normal subjects (median 2.13 versus 0.86ug/mL, *p* = 0.052) (Fig. 2). This difference was not observed across the entire cohort, and there was no difference in C3 in any age group by cognitive impairment. Finally, there were no significant differences in either CSF complement protein concentration across specific cognitive subdomains of impairment, as measured by domain-specific deficit scores (data not shown).

**Fig. 3:**
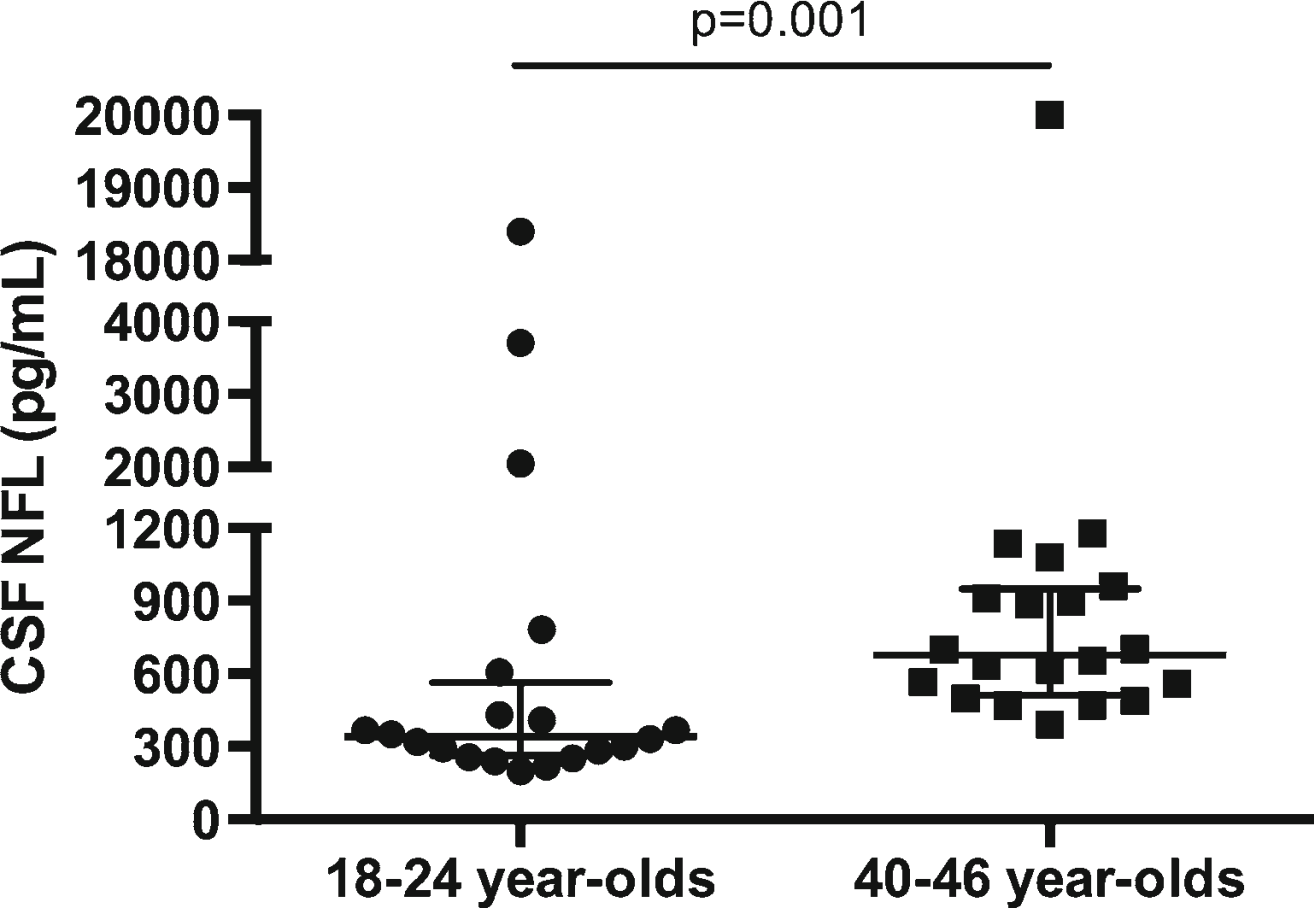
NFL was significantly lower in youth compared to older adults across the cohort (median 341 versus 677 pg/mL, *p* = 0.001)

There were no differences in C1q or C3 expression (in plasma or CSF) between older adults and youth, on or off ART (data not shown). NFL was significantly lower in youth compared to older adults across the cohort (median 341 versus 677 pg/mL, *p* = 0.001) (Fig. 3), consistent with prior reports in the literature that NFL is generally higher with advancing age (Jessen Krut et al. 2014). Furthermore, CSF NFL expression was inversely correlated with CD4+ T lymphocyte nadir (rho = −0.39, *p* = 0.013; data not shown), demonstrating a consistent relationship between systemic immune status and risk of CNS neuronal injury previously demonstrated in studies of older-aged HIV cohorts (Abdulle et al. 2007). Finally, CSF expression of C1q and C3 did not correlate with plasma expression C1q or C3, or clinical markers of systemic HIV disease progression, including CD4+ T lymphocyte nadir, current systemic CD4+ T lymphocyte count, or plasma/CSF viral load. These observations support the concept that CSF complement proteins are likely locally produced and regulated and not directly linked to peripheral immune dysregulation. Measuring complement levels in plasma moving forward is therefore likely not a good surrogate for CSF levels in clinical studies.

## Discussion

This is the first study to examine CSF complement protein expression as a novel immunologic mediator of HIV-associated neuronal damage in human subjects. It is also the first study to address the possibility of an age-specific ontogenetic neuropathogenesis of acquired HIV infection. We have demonstrated a potential positive correlation in this small cohort between CSF expression of C1q and NFL in subjects of all ages not receiving ART. These data suggest that C1q in CSF may be a correlate or biomarker for neuronal injury across the age span in HIV. Furthermore, in 18–24-year-old youth we found a nearly significant elevation of CSF C1q expression in cognitively impaired subjects to cognitively normal subjects. This second observation may reflect an age-specific vulnerability to complement-mediated cognitive dysfunction. Finally, we have confirmed a positive correlation between CSF expression of C1q and C3, suggesting our data are likely demonstrating true differences in activation of the complement system, rather than anomalous assay results. Borderline statistical significance may thus reflect actual biological significance but too small of a sample size for definitive results.

C1q is a 460 kDa hexameric glycoprotein that serves as a pattern recognition molecule and the first recognition subcomponent of the complement classical pathway. It binds HIV, IgG, IgM, and many other ligands and receptors largely via a gC1q domain (Kouser et al. 2015). It is locally produced in the CNS, likely by microglia, astrocytes, and neurons, and is generally upregulated during stress, injury, or infection. In addition to its roles in infection control, C1q plays a vital role with the complement cascade in normal synapse elimination during brain development (Stevens et al. 2007). Furthermore, in older adults, C1q has been associated with microglial activation and enhanced microglial-mediated phagocytosis, astrogliosis, and protection of neurons from β-amyloid and serum amyloid P-induced neurotoxicity (Kouser et al. 2015). It therefore balances function between neuroinflammation and neuroprotection depending on the context within which it is activated.

In the present study, we present preliminary evidence that C1q may be related to neurodegeneration. Whether that is via microglial activation and subsequent inflammatory excitotoxicity, or whether this relationship is simply a non-causal association is not yet clear. The fact that higher levels of C1q may be associated with cognitive impairment in youth suggest that C1q is likely at least a part of the causal pathway between HIV infection and neuronal damage, assuming neuronal damage is underlying cognitive dysfunction in HIV. This specificity of this finding in youth may reflect a biological relevance of normal complement developmental regulation in HIV neuropathogenesis. Specifically, synapses may be more vulnerable to a complement-mediated mechanism of neuronal destruction at certain developmental stages, perhaps due to a lack of local complement inhibition physiologically meant to permit normal developmental pruning processes.

Notably, while our sample size was low, C1q was not associated with systemic markers of HIV progression such as plasma HIV-1 RNA level (viral load) and CD4+ T lymphocyte count. This observation supports the idea that at least part of HIV neuropathophysiology occurs independently of peripheral disease and is clinically consistent with the fact that mild forms of HAND persist in the antiretroviral era in subjects with good systemic viral control.

There have been several recent compelling studies examining the presence of systemic inflammatory markers in youth with different developmental synaptopathies, including perinatally acquired HIV. One such study demonstrated an inverse correlation between serum markers of three combined pro-inflammatory markers (IL-6, C-reactive protein [CRP], fibrinogen) and processing speed, suggesting that residual inflammation may contribute to neurocognitive deficits despite well-controlled HIV disease in the periphery (Kapetanovic et al. 2014). While adolescents with behaviorally acquired HIV infection are a very different population in phenotype and viral pathogenesis than children with perinatal infection, these findings do raise interesting questions about the role of general systemic inflammation in HIV neuropathogenesis and how this may affect the CNS complement system. Biochemically, CRP can bind C1q and activate the classical complement cascade in the periphery. However, in the CNS, it is the mannose-binding lectin (MBL) pathway that is active, tagging synapses for microglial phagocytosis (Stevens et al. 2007). Thus, it is probably unlikely that CRP or other systemic pro-inflammatory cytokines are driving complement-mediated pathogenesis in the CNS. Instead, it is possible that inflammatory mediators in the periphery may help drive activated and infected monocytes into the CNS and thus perpetuate HIV neuropathogenesis (Fischer-Smith et al. 2008). Other inflammatory mediators known to be upregulated in the CSF of HIV+ individuals (e.g., monocyte chemoattractant protein (MCP)-1, beta-2-microglobulin, Substance P, IL-6, etc.) do not have an obvious direct link to complement activation.

Our study had several limitations. First, because we wanted to specifically include a comparable number of 18–22-year-old youth compared to older adults, the overall cohort size was relatively small (*n* = 40 total), because there are fewer specimens available from younger adults in the CHARTER cohort. Thus, overall analyses and sub-analyses with even smaller numbers (e.g., the relationship between C1q and NFL in subjects off ART) may be underpowered, and truly significant associations may therefore be underestimated. Second, while 19/36 subjects did have cognitive impairment (four were not classified), most of these (17/19) were mild (ANI). Therefore, linear correlations of complement markers with degree of impairment are limited. This is why we chose to use cognitive impairment as a binary variable in the majority of our analyses. Third, because of these small numbers and the cross-sectional nature of this correlation study, factors confounding the relationship between complement proteins and NFL are not accounted for in statistical associations. However, univariate analysis did not suggest relationships between complement proteins and systemic markers of disease, or peripheral complement levels, so not including these variables in subsequent models likely had little effect on the associations examined. In addition, causality cannot be inferred in a cross-sectional study. Finally, in our statistical analysis, we had several outlier values for NFL that we were not able to biologically explain why they otherwise differed from the remainder of the cohort. While the non-parametric analyses we used with our data should minimize the impact of outliers on statistical associations, a larger cohort would allow us in the future to determine if these subjects have a different specific biological reason for elevations in NFL, or if they were indeed spurious.

We chose to investigate the role of the complement system in HIV neuropathogenesis based on several recent articles by Stevens et al. (2007) and Stephan et al. (2012) reviewing the role of complement proteins in synaptic elimination and pruning. While HIV does not infect neurons, cognitive impairment in HIV is associated with pathological evidence of synaptic loss and dendritic simplification, as well as infection and activation of CNS infiltrating monocyte-derived macrophages and microglia (Masliah et al. 1997; Cherner et al. 2002; Ellis et al. 2007). Given the known interplay between the very complex complement system and HIV in the periphery, the question of the role of complement in HIV infection in the CNS is quite compelling. In summary, to date, we have demonstrated a tentative correlation between CSF expression of C1q and NFL in subjects not receiving ART across all ages, as well as a higher C1q expression associated with impaired neurocognitive function in 18–24 year old youth. These findings support the possibility of the complement system as a novel mediator of HIV neuropathogenesis and beg the question of whether a specific ontogenetic mechanism exists behind this neuropathogenesis. Future larger CSF studies and subsequent neuropathological studies are necessary to determine if these relationships are maintained in larger cohorts and tissue specimens. If so, these finding may have future therapeutic implications for novel mechanisms of adjunctive, age-specific HAND therapies (e.g. complement inhibitors).

## References

[CR1] Abdulle S, Mellgren A, Brew BJ (2007). CSF neurofilament protein (NFL)—a marker of active HIV-related neurodegeneration. J Neurol.

[CR2] Alexander JJ, Anderson AJ, Barnum SR (2008). The complement cascade: Yin-Yang in neuroinflammation—neuro-protection and -degeneration. J Neurochem.

[CR3] Antinori A, Arendt G, Becker JT (2007). Updated research nosology for HIV-associated neurocognitive disorders. Neurology.

[CR4] Carey CL, Woods SP, Gonzalez R (2004). Predictive validity of global deficit scores in detecting neuropsychological impairment in HIV infection. J Clin Exp Neuropsychol.

[CR5] Centers for Disease Control and Prevention (2015) HIV among youth. In: cdc.gov. http://www.cdc.gov/hiv/group/age/youth/. Accessed 30 Nov 2015

[CR6] Cherner M, Masliah E, Ellis RJ (2002). Neurocognitive dysfunction predicts postmortem findings of HIV encephalitis. Neurology.

[CR7] Ellis R, Langford D, Masliah E (2007). HIV and antiretroviral therapy in the brain: neuronal injury and repair. Nat Rev Neurosci.

[CR8] Fischer-Smith T, Bell C, Croul S (2008). Monocyte/macrophage trafficking in acquired immunodeficiency syndrome encephalitis: lessons from human and nonhuman primate studies. J Neurovirol.

[CR9] Gelman BB, Chen T, Lisinicchia JG (2012). The national NeuroAIDS tissue consortium brain gene array: two types of HIV-associated neurocognitive impairment. PLoS ONE.

[CR10] Giedd JN, Blumenthal J, Jeffries NO (1999). Brain development during childhood and adolescence: a longitudinal MRI study. Nat Neurosci.

[CR11] Gisslen M, Hagberg L, Brew BJ (2007). Elevated cerebrospinal fluid neurofilament light protein concentrations predict the development of AIDS dementia complex. J Infect Dis.

[CR12] Gresle MM, Butzkueven H, Shaw G (2011). Neurofilament proteins as body fluid biomarkers of neurodegeneration in multiple sclerosis. Mult Scler Int.

[CR13] Heaton RK, Clifford DB, Franklin DR (2010). HIV-associated neurocognitive disorders persist in the era of potent antiretroviral therapy: CHARTER study. Neurology.

[CR14] Jessen Krut J, Mellberg T, Price RW (2014). Biomarker evidence of axonal injury in neuroasymptomatic HIV-1 patients. PLoS ONE.

[CR15] Jongen PJ, Doesburg WH, Ibrahim-Stappers JL (2000). Cerebrospinal fluid C3 and C4 indexes in immunological disorders of the central nervous system. Acta Neurol Scand.

[CR16] Julien JP (1999). Neurofilament functions in health and disease. Curr Opin Neurobiol.

[CR17] Kapetanovic S, Griner R, Zeldow B (2014). Biomarkers and neurodevelopment in perinatally HIV-infected or exposed youth: a structural equation model analysis. AIDS.

[CR18] Kouser L, Madhukaran SP, Shastri A (2015). Emerging and novel functions of complement protein C1q. Front Immunol.

[CR19] Liu F, Dai S, Gordon J, Qin X (2014). Complement and HIV-I infection/HIV-associated neurocognitive disorders. J Neurovirol.

[CR20] Masliah E, Heaton RK, Marcotte TD (1997). Dendritic injury is a pathological substrate for human immunodeficiency virus-related cognitive disorders.. Ann Neurol.

[CR21] McGuire JL, Gill AJ, Douglas SD (2015). Central and peripheral markers of neurodegeneration and monocyte activation in HIV-associated neurocognitive disorders. J Neurovirol.

[CR22] Mellgren A, Price RW, Hagberg L (2007). Antiretroviral treatment reduces increased CSF neurofilament protein (NFL) in HIV-1 infection. Neurology.

[CR23] Pasol J, Feuer W, Yang C (2010). Phosphorylated neurofilament heavy chain correlations to visual function, optical coherence tomography, and treatment. Mult Scler Int.

[CR24] Peluso MJ, Meyerhoff DJ, Price RW (2013). Cerebrospinal fluid and neuroimaging biomarker abnormalities suggest early neurological injury in a subset of individuals during primary HIV infection. J Infect Dis.

[CR25] Schafer DP, Lehrman EK, Kautzman AG (2012). Microglia sculpt postnatal neural circuits in an activity and complement-dependent manner. Neuron.

[CR26] Speth C, Williams K, Hagleitner M (2004). Complement synthesis and activation in the brain of SIV-infected monkeys. J Neuroimmunol.

[CR27] Stephan AH, Barres BA, Stevens B (2012). The complement system: an unexpected role in synaptic pruning during development and disease. Annu Rev Neurosci.

[CR28] Stevens B, Allen NJ, Vazquez LE (2007). The classical complement cascade mediates CNS synapse elimination. Cell.

[CR29] Woods SP, Rippeth JD, Frol AB (2004). Interrater reliability of clinical ratings and neurocognitive diagnoses in HIV. J Clin Exp Neuropsychol.

